# Antiproliferative and Apoptosis-Inducing Activities of 4-Isopropyl-2,6-bis(1-phenylethyl)phenol Isolated from Butanol Fraction of *Cordyceps bassiana*

**DOI:** 10.1155/2015/739874

**Published:** 2015-04-02

**Authors:** Ji Hye Kim, Yunmi Lee, Gi-Ho Sung, Han Gyung Kim, Deok Jeong, Jae Gwang Park, Kwang-Soo Baek, Nak Yoon Sung, Sungjae Yang, Deok Hyo Yoon, Sang Yeol Lee, Hyojeung Kang, Changsik Song, Jae Han Cho, Kang-Hyo Lee, Tae Woong Kim, Jae Youl Cho

**Affiliations:** ^1^Department of Genetic Engineering, Sungkyunkwan University, Suwon 440-746, Republic of Korea; ^2^Department of Chemistry, Kwangwoon University, Seoul 139-701, Republic of Korea; ^3^Institute for Bio-Medical Convergence, International St. Mary's Hospital and College of Medicine, Catholic Kwandong University, Incheon 404-834, Republic of Korea; ^4^Department of Biochemistry, Kangwon National University, Chuncheon 220-700, Republic of Korea; ^5^Department of Life Science, Gachon University, Seongnam, Kyeonggi-do 461-701, Republic of Korea; ^6^College of Pharmacy and Research Institute of Pharmaceutical Sciences, Kyungpook National University, Daegu 702-701, Republic of Korea; ^7^Department of Chemistry, Sungkyunkwan University, Suwon 440-746, Republic of Korea; ^8^Mushroom Research Division, Department of Herbal Crop Research, National Institute of Horticultural & Herbal Science, RDA, Suwon 441-707, Republic of Korea; ^9^Department of Biochemistry, Kangwon National University, Chuncheon 200-701, Republic of Korea

## Abstract

The *Cordyceps* species have been widely used for treating various cancer diseases. Although the Cordyceps species have been widely known as an alternative anticancer remedy, which compounds are responsible for their anticancer activity is not fully understood. In this study, therefore, we examined the anticancer activity of 5 isolated compounds derived from the butanol fraction (Cb-BF) of *Cordyceps bassiana*. For this purpose, several cancer cell lines such as C6 glioma, MDA-MB-231, and A549 cells were employed and details of anticancer mechanism were further investigated. Of 5 compounds isolated by activity-guided fractionation from BF of Cb-EE, KTH-13, and 4-isopropyl-2,6-bis(1-phenylethyl)phenol, Cb-BF was found to be the most potent antiproliferative inhibitor of C6 glioma and MDA-MB-231 cell growth. KTH-13 treatment increased DNA laddering, upregulated the level of Annexin V positive cells, and altered morphological changes of C6 glioma and MDA-MB-231 cells. In addition, KTH-13 increased the levels of caspase 3, caspase 7, and caspase 9 cleaved forms as well as the protein level of Bax but not Bcl-2. It was also found that the phosphorylation of AKT and p85/PI3K was also clearly reduced by KTH-13 exposure. Therefore, our results suggest KTH-13 can act as a potent antiproliferative and apoptosis-inducing component from *Cordyceps bassiana*, contributing to the anticancer activity of this mushroom.

## 1. Introduction

The* Cordyceps* species are a representative of insect-born mushrooms which have been prescribed as well-known traditional herbal medicines in Korea, China, and Japan [1]. These mushrooms are ethnopharmacologically known to enhance longevity, endurance, and vitality for normal healthy people, but also to ameliorate various human diseases such as skin diseases, chronic bronchitis, asthma, and tuberculosis [1, 2]. Through systematic studies, it has been revealed that this mushroom helps expanding of life span of cancer patients by displaying direct anticancer activities. In addition, other numerous pharmacological activities, such as antioxidative, antiviral, antifibrotic, anti-inflammatory, antinociceptive, antiangiogenic, antiplatelet aggregation, and antidiabetic effect, stress the significant medicinal value of this mushroom [2–5].

**Figure 1:**
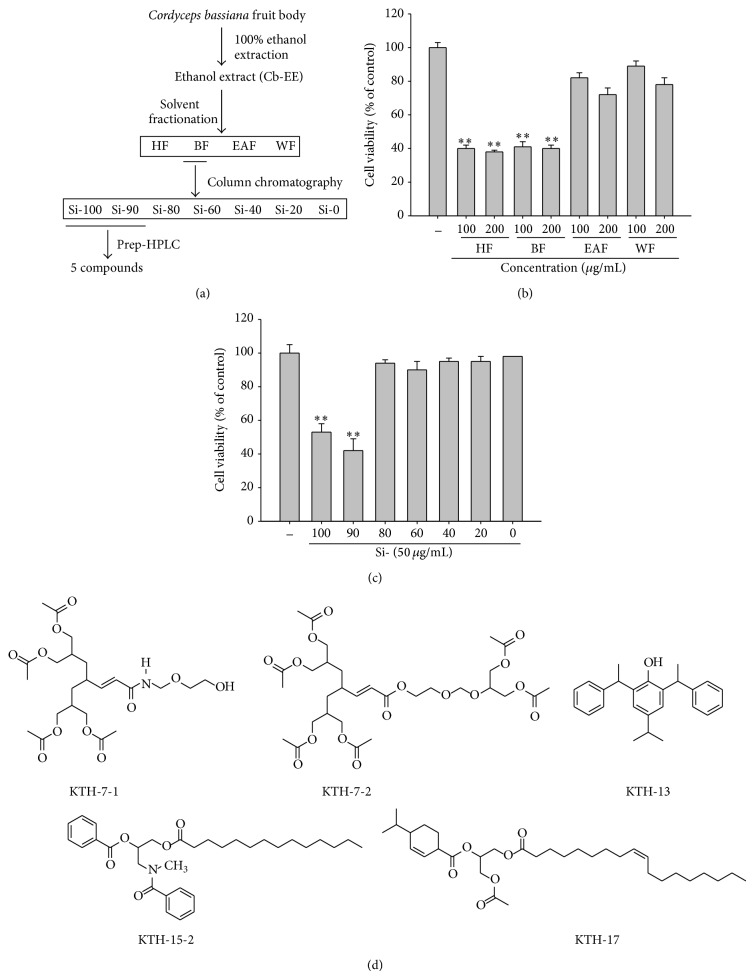
Schematic isolation procedure of 5 compounds by activity-guided fractionation and their chemical structures. (a) Schematic procedure of isolation strategy of 5 compounds from fruit bodies of* Cordyceps bassiana*. (b and c) Antiproliferative activities of fractions treated for 24 h in A549 cells were tested by a conventional MTT assay. (d) Chemical structures of 5 isolated compounds. All of the data are expressed as the means ± SD of experiments that were performed with six samples. ^∗∗^ *P* < 0.01, as compared to the vehicle control.

So far, only few compounds with anticancer activities have been identified from* Cordyceps* species. These compounds include cordycepin isolated from* Cordyceps militaris*, and CME-1, a water-soluble polysaccharide fraction from* Cordyceps sinensis* mycelia. Although some polysaccharides (e.g., *β*-glucans) are reported to enhance an anticancer activity of innate immunity, studies on those compounds with direct anticancer activities have not yet been fully elucidated. In this study, we evaluated anticancer activity of a newly identified compound, KTH-13 [4-isopropyl-2,6-bis(1-phenylethyl)phenol (Figure 1)], from artificially cultivated fruit bodies of* Cordyceps bassiana* through activity-guided fractionation. To check whether this compound is able to directly suppress the viability of cancer cells, we tested its antiproliferative activity and investigated the anticancer mechanism by characterization of its proapoptotic pathway.

## 2. Materials and Methods

### 2.1. Materials

*Cordyceps bassiana* identified by Professor Jae Mo Sung (Kangwon National University, Chuncheon, Korea) was obtained from Mush-Tech (Chuncheon, Korea). A voucher specimen of this (number 278-Cb-1) was deposited in the herbarium of our laboratory. (3-4,5-Dimethylthiazol-2-yl)-2,5-diphenyltetrazolium bromide, tetrazole (MTT), propidium iodide (PI), and staurosporine were purchased from Sigma Chemical Co. (St. Louis, MO, USA). A FITC Annexin V Apoptosis Detection Kit was from eBioscience (San Diego, CA, USA). Foetal bovine serum (FBS) and RPMI1640 were obtained from GIBCO (Grand Island, NY, USA). C6 glioma, MDA-MB-231, and A549 cells were purchased from ATCC (Rockville, MD, USA). All other chemicals were purchased from Sigma. Phosphospecific and total antibodies against caspases (3, 7, 8, and 9), Bax, Bcl-2, AKT, p85/PI3K, mTOR, Src, p65, and *β*-actin were obtained from Cell Signalling (Beverly, MA, USA).

### 2.2. General Experimental Procedures

All of the NMR spectra were measured on a Varian UNITY INOVA 500 spectrometer or on a Bruker AMX 500 spectrometer operating at 500 MHz for ^1^H-NMR and 125 MHz for ^13^C-NMR in DMSO-*d*_6_ using TMS as an internal standard. EI-MS was measured on a Hewlett Packard model 5989B GC/MS spectrometer. The HPLC apparatus (Waters Alliance series 2795 system) was composed of a vacuum degasser, a quaternary pump, a photodiode array detector (PDA), an autoinjector, and a column compartment with a thermostat. A Luna 5 *μ* C18(2) 100 A column (4.6 × 150 mm 5 *μ*m, Phenomenex) was used for isolation and purification of the compounds. A silica gel (70–230 mesh, Merck) and a Sephadex LH-20 (GE Healthcare) were used for open column chromatography. TLC was performed on silica gel (60 F_254_, Merck).

### 2.3. Extraction and Isolation

Dried and powdered fresh fruiting bodies of* C. bassiana* (3.0 kg) were extracted with 100% EtOH to prepare the Cb-EE. After removing the polysaccharide layer and performing lyophilisation, the powder was then reextracted with n-hexane, n-butanol, ethyl acetate (three times, each with 250 mL) subsequently to obtain hexane fraction (HF), butanol fraction (BF), and ethyl acetate fraction (EAF), by using a reflux apparatus upon removal of the solvent* in vacuo*. Of subfractions, BF was separated by silica gel column chromatography and eluted with CHCl_3_-MeOH mixtures of increasing polarity (100 : 0→20 : 0) to afford seven fractions (Si-0~Si-100). Mixed fractions of Si-90 and Si-100 were purified by reverse phase C18 column chromatography using CH_3_CN in H_2_O to produce 5 compounds [KTH-7-1: (E)-2-(2-(3-acetoxy-2-(acetoxymethyl)propyl)-5-(((2-hydroxyethoxy) methyl)amino)-5-oxopent-3-en-1-yl)propane-1,3-diyl diacetate (Bassiamide A), KTH-7-2: (E)-2-(15-(3-acetoxy-2-(acetoxymethyl)propyl)-5-(acetoxymethyl)-2,12-dioxo-3,6,8,11-tetraoxahexadec-13-en-16-yl)propane-1,3-diyl diacetate (Bassiamate), KTH-13: 4-isopropyl-2,6-bis(1-phenylethyl)phenol (IPr-PEPhenol), KTH-15-2: 1-(N-methylbenzamido)-3-(tetradecanoyloxy)propan-2-yl benzoate, and KTH-17: (Z)-1-acetoxy-3-(oleoyloxy)propan-2-yl 4-isopropylcyclohex-2-enecarboxylate (AOGTE)]. To identify chemical structures of these compounds, additional phytochemical studies were continuously carried out, as reported previously [6]. Their physicochemical and spectroscopic data are included in supplementary figures. (See Supplementary Material available online at http://dx.doi.org/10.1155/2015/739874).

### 2.4. Cell Culture

C6 glioma, MDA-MB-231, and A549 cells were cultured in RPMI 1640 medium supplemented with 10% heat-inactivated FBS, glutamine, and antibiotics (penicillin and streptomycin) at 37°C in 5% CO_2_. For each experiment, cells were detached with trypsin/EDTA solution. At the cell density used in our experiments (2 × 10^6^ cells/mL), the proportion of dead cells was less than 1% according to Trypan blue dye exclusion tests.

### 2.5. Cell Viability Test

After preincubation of C6 glioma, MDA-MB-231, and A549 cells (1 × 10^6^ cells/mL) for 18 h, testing compounds or fractions were added to the cells and incubated for 24 or 48 h under 2.5% FBS conditions. The cytotoxic effect of KTH-13 was then evaluated by a conventional MTT assay, as previously reported [7, 8]. Three hours prior to culture termination, 10 *μ*L of MTT solution (10 mg/mL in phosphate-buffered saline (PBS), pH 7.4) was added to the cultures, and the cells were continuously cultured until termination of the experiment. The incubation was halted by the addition of 15% sodium dodecyl sulphate to each well to solubilise the formazan [9]. The absorbance at 570 nm (OD_570–630_) was measured using a Spectramax 250 microplate reader.

### 2.6. DNA Fragmentation Assay

DNA was isolated using GeneAll assay kit (Seoul, Korea) according to the manufacturer's protocol [10]. Briefly KTH-13- or staurosporine-treated cells were pelleted and were lysed by adding proteinase K and 200 *μ*L of lysis buffer provided in the kit. The samples were incubated at 56°C until complete lysis. 200 *μ*L of lysis buffer was again added and incubated at 70°C for 10 min. After adding absolute ethanol to the samples, the mixture was transferred to SV column and centrifuge at 6000 rpm to collect the DNA. The column was washed twice with washing buffer and centrifuged at full speed to dry the membrane and remove residual ethanol which may interfere with subsequent reactions. DNA was eluted in 50 *μ*L of elution buffer supplied with the kit and collected in fresh Eppendorf tube. The DNA samples were then subjected to 1.5% agarose gel electrophoresis at 100 V for 2.5 h at room temperature. Tris acetate EDTA was used as the running buffer and DNA bands were visualized under UV light.

### 2.7. Annexin V-PI Staining Apoptosis Assay

Apoptosis was determined using FITC Annexin V Apoptosis Detection Kit based on the membrane changes (phosphatidylserine based) [11, 12]. Cells were plated in 60 mm culture dish at a seeding density of 5 × 10^5^ cells/dish and KTH-13 was added to the culture media to the specified final concentration. Vehicle was added alone to the culture medium serving as the untreated control. The subsequent procedures were carried out according to the instructions provided by the manufacturer. Briefly, after 24 h, cells were harvested, washed twice with PBS, and resuspended in 1X binding buffer. Annexin-V FITC and PI were added and incubated for 15 min at room temperature (25°C) in the dark. Fluorescence from a population of 1 × 10^5^ cells was detected using the BD FACSCan flow cytometer (Becton Dickenson, Mountain View, CA, USA) and CellQuest Pro (IVD) software (Becton Dickenson, Mountain View, CA, USA). The assays were done in duplicate and repeated in three independent experiments.

### 2.8. Morphological Change Test

KTH-13-treated C6 glioma and MDA-MB-231 cells were incubated for indicated times. Images of the cells in culture at each time point were obtained using an inverted phase contrast microscope, attached to a video camera, and captured using NIH image software as reported previously [9].

### 2.9. Preparation of Cell Lysates and Immunoblotting Analysis

C6 glioma cells (5 × 10^6^ cells/mL) were washed three times in cold PBS containing 1 mM sodium orthovanadate and then lysed in lysis buffer (20 mM Tris-HCl, pH 7.4, 2 mM EDTA, 2 mM ethyleneglycotetraacetic acid, 50 mM *β*-glycerophosphate, 1 mM sodium orthovanadate, 1 mM dithiothreitol, 1% Triton X-100, 10% glycerol, 10 *μ*g/mL aprotinin, 10 *μ*g/mL pepstatin, 1 mM benzimide, and 2 mM PMSF) for 30 min with rotation at 4°C. The lysates were clarified by centrifugation at 16,000 ×g for 10 min at 4°C and then stored at −20°C until needed.

Whole cells were then analyzed using immunoblotting [13, 14]. Proteins were separated on 10% SDS-polyacrylamide gels and transferred by electroblotting onto a polyvinylidene difluoride (PVDF) membrane. Membranes were blocked for 60 min in Tris-buffered saline containing 3% FBS, 20 mM NaF, 2 mM EDTA, and 0.2% Tween 20 at room temperature. The membranes were incubated for 60 min with specific primary antibodies at 4°C, washed three times with the same buffer, and then incubated for an additional 60 min with HRP-conjugated secondary antibodies. The total and phosphorylated levels of the signaling enzymes and transcription factors were visualized using an ECL system (Amersham, Little Chalfont, Buckinghamshire, UK), as previously reported.

### 2.10. Statistical Analysis

All data presented in this paper are the mean ± standard deviation (SD) of an experiment performed with six samples. For statistical comparisons, the results were analysed using ANOVA/Scheffe's post hoc test and Kruskal-Wallis/Mann-Whitney tests. A *P* value <0.05 was considered statistically significant. All statistical tests were performed using SPSS software (SPSS Inc., Chicago, IL, USA). Similar experimental data were also obtained in an additional independent set of experiments performed with the same number of samples.

## 3. Results and Discussion

Anticancer activities of* Cordyceps* have already been reported previously. Thus, it was reported that the hot water extracts of* Cordyceps militaris* cultured mycelia (CMMY) and cultivated fruiting bodies (CMFB) are able to kill various human leukemia cells [15]. The proliferation of cancer cell lines, HepG2 (liver), MCF-7 (breast), and A549 (lung) was found to be suppressed by culture medium of* Cordyceps sinensis* with polyphenols and flavonoids [16]. Jiangxienone, isolated from a culture of* Cordyceps jiangxiensis*, has been reported to suppress the proliferation of human gastric adenocarcinoma SGC-7901 cells and human lung carcinoma A549 cells with a potency of IC_50_ values ranging from 1.38 to 2.93 *μ*M [17]. In addition,* Cordyceps pruinosa* butanol fraction was also revealed to induce cytotoxic activity against HeLa cells via the upregulation of apoptosis [18]. These results imply a possibility that* Cordyceps* species generally have antiproliferative and proapoptotic activities. To test this hypothesis, we aimed to investigate whether the artificially cultivated* Cordyceps bassiana* fruit bodies and their chemical ingredients are capable of lowering the viability of cancer cells by demonstrating their effects on various cancer cell lines, including rat C3 glioma cells and human breast cancer MDA-MB-231 cells.

As shown in other* Cordyceps* species [19], butanol (BF) and hexane (HF) fractions exhibited strong antiproliferative activities at 100 and 200 *μ*g/mL (Figure 1(b)) of solvent fractions with variable yields (Figure 1, Table 1, and Supplementary Figure 1). After considering the chemical property of other previous studies performed with* Cordyceps pruinosa* [18], we further fractionated BF by silica gel column chromatography to prepare another 7 subfractions (Figure 1(a)). In fact, the cytotoxic activity of BF was shown in fractions Si-90 and -100 (Figure 1(c) and Supplementary Figure 2). Through an additional fractionation process by prep-HPLC, we were finally able to identify 5 compounds (Figure 1(d)) from the peaks in the chromatogram (see Supplementary Figure 3).

**Figure 2:**
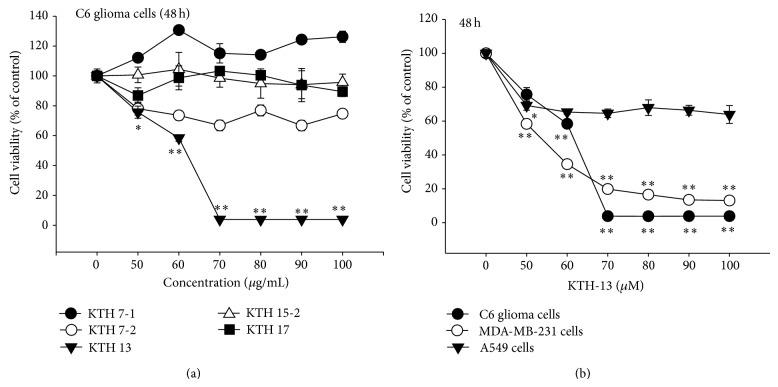
The effects of 5 isolated compounds on the cell proliferation of various cancer cells (MDA-MB-231 cells, C6 glioma, and A549 cells). (a) Antiproliferative activities of KTHs 7-1, 7-2, 13, 15-2, and 17 on the proliferation of C6 glioma cells were determined by MTT assay. (b) MDA-MB-231 cells, C6 glioma, and A549 cells (1 × 10^6^ cells/mL) were incubated with KTH-13 for 48 h. Cell viability was determined by conventional MTT assay. All of the data are expressed as the means ± SD of experiments that were performed with six samples. ^∗^ *P* < 0.05 and ^∗∗^ *P* < 0.01, as compared to the vehicle control.

Next, we tested whether the isolated compounds are able to suppress the viability of cancer cells. As Figure 2(a) shows, of the 5 compounds, KTH-13 exhibited a strong antiproliferative activity against C6 glioma cells. Through continuous tests with other cell lines, it was also found that the viability of MDA-MB-231 cells was also dose-dependently suppressed by this compound. The IC_50_ values of KTH-13 with 50 to 60 *μ*M were summarized in Tables 2 and 3. These results, therefore, drew us into a further investigation of the compound's anticancer mechanism.

**Figure 3:**
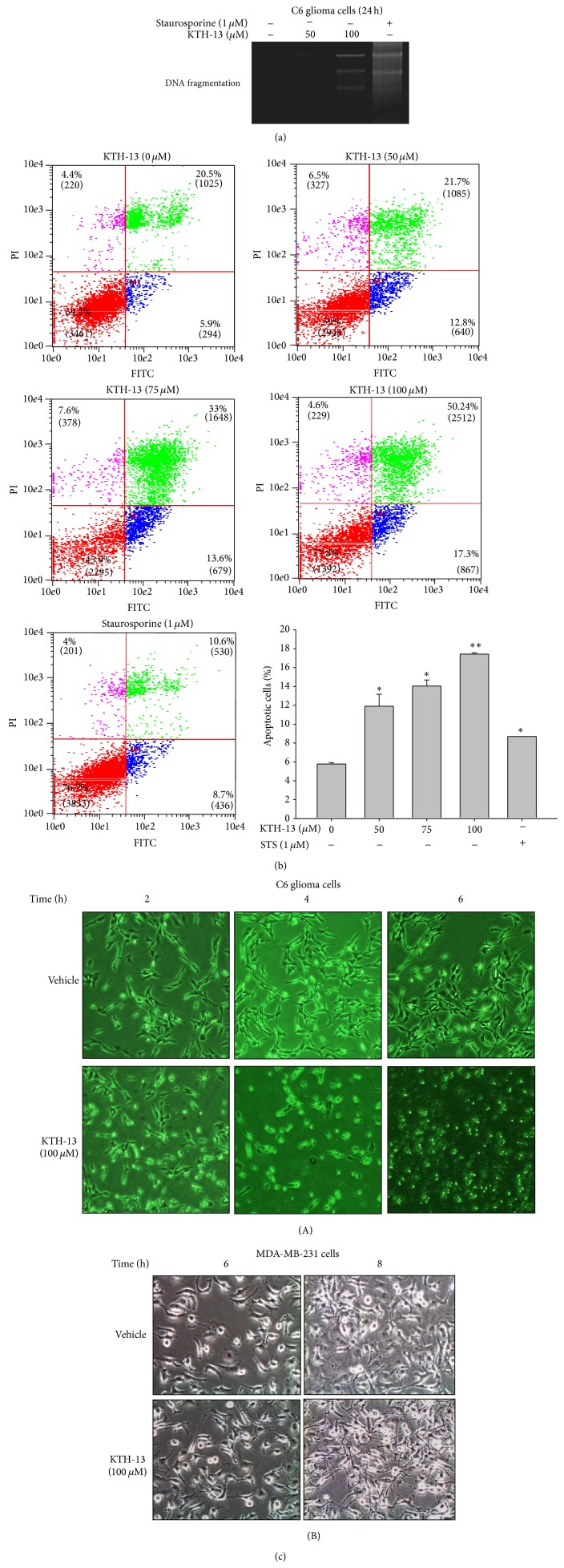
Apoptosis-inducing effects of KTH-13 in C6 glioma cells. (a) C6 glioma cells (5 × 10^6^ cells/mL) were incubated with KTH-13 or staurosporine (STS) for 24 h. After preparing DNA extracts, DNA laddering patterns were evaluated by agarose gel electrophoresis. (b) Cell apoptosis was determined by Annexin V-FITC and PI double-staining analysis. The KTH-13- or STS-treated apoptotic cells with Annexin V and PI treatment were analyzed by flow cytometry. Images from three experiments are shown. (c) Morphological changes of C6 glioma (A) and MDA-MB-231 (B) cells induced by KTH-13 were observed by microscopic analysis. All of the data were obtained from one of three independent experiments.

To further confirm whether the suppressive activity of KTH-13 is generated by proapoptotic or pronecrotic activity of this compound, we next examined the pattern of DNA fragmentations.  Figure 3(a) depicts similar patterns of DNA ladders between KTH-13 and a control compound, staurosporine, treated for 24 h, indicating that both KTH-13 and staurosporine are able to induce apoptotic processes in C6 glioma, in which the latter is previously reported [20]. In agreement with this result (Figure 3(a)), C6 glioma cells stained with Annexin V-FITC, an early apoptotic marker [21], displayed a dose-dependent increase pattern of FITC level from 5.8 to 17.4% at 50 to 100 *μ*M of KTH-13 (Figure 3(b)). Under the same conditions, standard compound, staurosporine (STS), also significantly increased the numbers of apoptotic cells (Figure 3(b)). More interestingly, KTH-13 triggered actin cytoskeleton-dependent morphological changes in MDA-MB-231 cells and C6 glioma cells (Figure 3(c)). Such occurrences induced by KTH-13 seem to be accompanied when cells are differentiated and apoptotic, which linked to growth arrest and cell death [22, 23].

**Figure 4:**
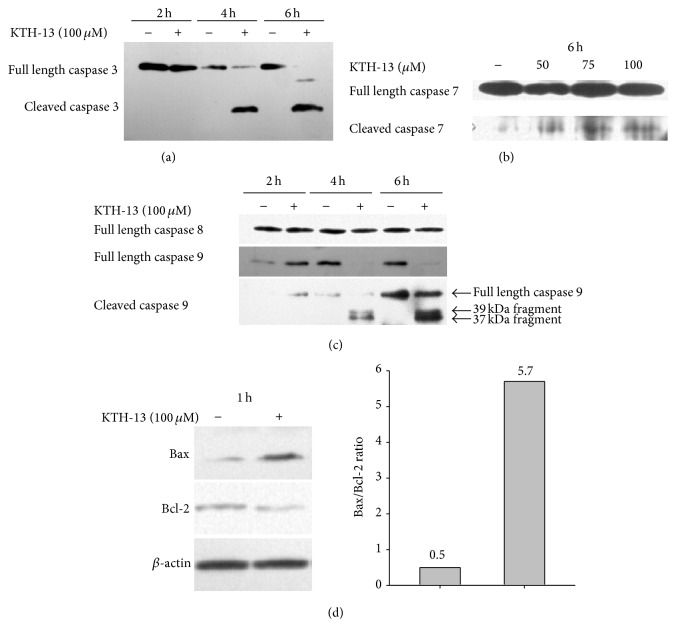
The effect of KTH-13 on the levels of proapoptotic and antiapoptotic proteins. (a, b, and c) C6 glioma cells (5 × 10^6^ cells/mL) were incubated with KTH-13 for the indicated times. After immunoblotting analysis with whole cell lysates, total or cleaved levels of caspases 3, 7, 8, and 9 were identified with specific antibodies. (d) C6 glioma cells (5 × 10^6^ cells/mL) were incubated with KTH-13 for 1 h. The levels of Bax and Bcl-2 from whole cell lysates were analyzed by their specific antibody. All of the data were obtained from one of three independent experiments.

Since KTH-13 seems to induce apoptosis of C6 glioma cells, we next examined the molecular mechanism of its proapoptotic activity by measuring cleaved caspase patterns. First, whether the KTH-13-induced apoptosis is generated by either a death receptor-mediated extrinsic pathway or a mitochondrial-dependent intrinsic pathway was determined by identification of caspase 8 or 9. As Figure 4(a) shows, it was revealed that KTH-13-induced apoptotic activity might be mediated by mitochondrial pathway. Thus, this compound upregulated the cleaved form of caspase 3 (Figure 4(a)), caspase 7 (Figure 4(b)), and caspase 9 (Figure 4(c)). However, the level of full length caspase 8 was not altered by KTH-13, indicating that this compound does not affect caspase 8-dependent apoptotic pathway (Figure 4(c)). In addition, KTH-13 triggered the upregulation of Bax and downregulation of Bcl-2 (Figure 4(d)), required for the complex formation between Apaf-1 and procaspase 9 to activate caspase 9 [24]. Therefore, these results strongly suggest that KTH-13 is able to induce proapoptotic signaling via provoking mitochondrial-dependent intrinsic pathway. Meanwhile, it was reported that cordycepin-induced apoptotic pathway is not accompanied with an increased level of cleaved caspase 3 in C6 glioma cells [25]. Cordycepol C, a novel sesquiterpene isolated from the cultured mycelia of Cordyceps* ophioglossoides*, was also known to trigger caspase-independent apoptosis in HepG2 cells [26]. These prior reports could indicate that KTH-13-mediated apoptosis might be distinct from that of cordycepin and cordycepol C.

**Figure 5:**
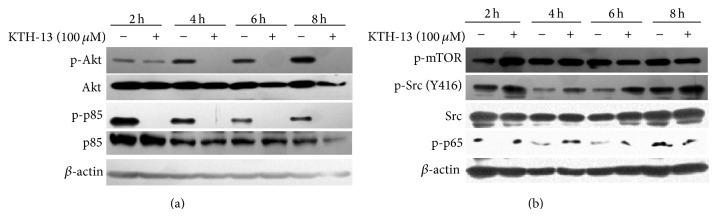
The effect of KTH-13 on the levels of cell survival signaling proteins. (a and b) C6 glioma cells (5 × 10^6^ cells/mL) were incubated with KTH-13 for the indicated times. After immunoblotting with whole cell lysates, total or phospholevels of AKT, p85/PI3K, Src, mTOR, p65, and *β*-actin were identified with specific antibodies. All of the data were obtained from one of three independent experiments.

Because cell survival signaling is also linked to regulation of proapoptotic signaling, we finally examined whether KTH-13 can also regulate cell survival signaling cascade. As Figure 5(a) shows, interestingly, this compound strongly suppressed the phosphorylation of AKT and its upstream enzyme p85/PI3K, implying that KTH-13 can also modulate AKT-mediated survival signaling. However, the facts that the phosphorylation of mTOR, a downstream enzyme phosphorylated by AKT, and Src phosphorylation, an upstream signaling enzyme contributed to AKT activation, were not suppressed (Figure 5(b)) strongly indicate that KTH-13-mediated inhibition of PI3K/AKT might be limited to heightening proapoptotic signaling managed by caspase activity. Indeed, it was reported that PI3K/AKT pathway is involved in suppression of caspase 3 activity [27]. Thus, it is likely that PI3K/AKT inhibition by KTH-13 could affect the activation of caspase 3. However, since details of relationship between AKT inhibition and mitochondrial damage-induced apoptotic pathway remain unclear, our future works will be focused on verifying such cross regulation by KTH-13.

**Figure 6:**
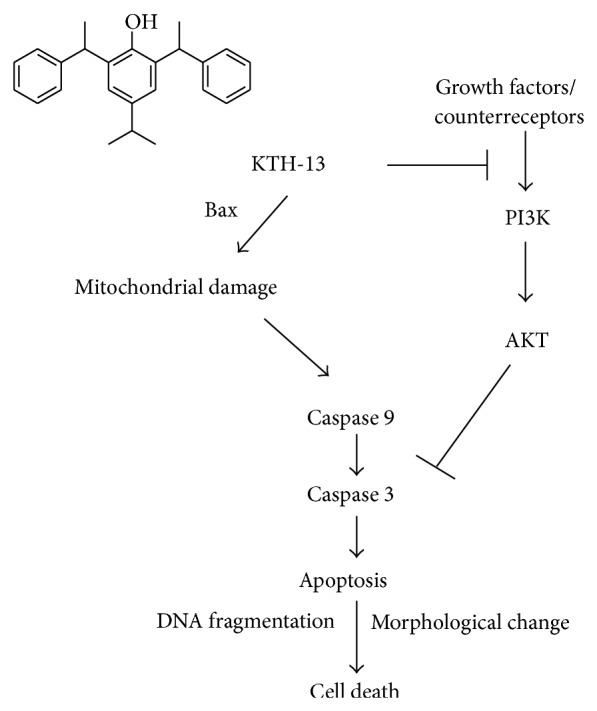
Putative apoptosis-inducing mechanism of KTH-13 in cancer cells.

In conclusion, we demonstrated that, of the 5 isolated compounds by activity-guided fractionation from BF of Cb-EE, KTH-13 was found as the strongest inhibitor of C6 glioma and MDA-MB-231 cell viability. The growth inhibitory activity of KTH-13 was due to proapoptotic activity managed by mitochondria-dependent intrinsic pathway composed of caspases 9 and 3, as well as the inhibition of PI3K/AKT, as summarized in Figure 6. Therefore, our results suggest the value of* Cordyceps bassiana* and propose the putative use of KTH-13 isolated from this mushroom as the main component of* Cordyceps bassiana*-containing anticancer remedy. Since we have already established a total synthetic procedure for preparing KTH-13 (data not shown), we will also further derivatize this compound to maximize its anticancer potency. Finally, by exploring additional* in vivo* experiments, we will also confirm whether KTH-13 preserves its effective anticancer activity when orally administered.

## Supplementary Material

Supplementary Fig. 1. Fractionation procedure to prepare solvent fractions by sequential solvent fractionation and thin layer chromatography profiles of *C. bassiana* ethanol extract (1) and its sub-fractions (5 to 8). The crude ethanol extract was subfractinated by sequential fractionation procedure with various solvents to afford eight sub-fractions. Ingredients in these fractions were also detected by thin layer chromatography.
Supplementary Fig. 2. Fractionation procedure to prepare fractions by silica column chromatography and thin layer chromatography profiles of these fractions at 254 and 366 nm. The fractions were chromatographed over a silica gel column to afford seven sub-fractions. Ingredients in these fractions were detected by thin layer chromatography.
Supplementary Fig. 3. Isolated compounds from peaks separated by prep-HPLC. Ingredient compounds displaying each peak in each fraction were purified by preparative HPLC. The structure of isolated compounds in each peak was identified by spectroscopic analysis.
Supplementary Fig. 4. Spectroscopic profile of KTH-13. (A) Mass spectrum of KTH-13. Electrospray ionization tandem mass spectrometry (ESI MS) of KTH-13 was measured on an Agilent 1100 liquid chromatography/mass spectrometry (LC/MS) spectrometer with a Phenomenex Luna C18 analytical column. (B) 1H-NMR profile of KTH-13. 1H-NMR spectra of KTH-13 were recorded on a Bruker Avance 300 (300 MHz) and Bruker DPX 400 (400 MHz). Chemical shifts are reported in parts per million (ppm) downfield relative to tetramethylsilane as an internal standard.
Supplementary Fig. 5. Mass spectrum of KTH-7-1. ESI MS of KTH-7-1 was measured on an Agilent 1100 LC/MS spectrometer with a Phenomenex Luna C18 analytical column.
Supplementary Fig. 6. Mass spectrum of KTH-7-2. ESI MS of KTH-7-2 was measured on an Agilent 1100 LC/MS spectrometer with a Phenomenex Luna C18 analytical column.
Supplementary Fig. 7. Mass spectrum of KTH-15-2. ESI MS of KTH-15-2 was measured on an Agilent 1100 LC/MS spectrometer with a Phenomenex Luna C18 analytical column.
Supplementary Fig. 8. Mass spectrum of KTH-17. ESI MS of KTH-17 was measured on an Agilent 1100 LC/MS spectrometer with a Phenomenex Luna C18 analytical column.

## Figures and Tables

**Table 1:** Yield of solvent extracts from artificially cultivated *Cordyceps  bassiana* fruit bodies.

Sample	Weight (g)	Yield (%)
Fruit bodies	3,000	
Ethanol extract	533.7	17.8
Hexane fraction (HF)	87.3	2.9
Butanol fraction (BF)	72.5	2.4
Ethyl acetate fraction (EAF)	1.2	0.04
Water fraction (WF)	336.6	11.2

**Table 2:** Inhibitory effect of compounds isolated from BF of Cb-EE on the proliferation of C6 glioma cells.

Compound	IC_50_ (*μ*M)
KTH-7-1	>100
KTH-7-2	>100
KTH-13	64.9 ± 1.3
KTH-15-2	>100
KTH-17	>100

**Table 3:** Inhibitory effect of KTH-13 on the proliferation of C6 glioma, MDA-MB-231, and A549 cells.

Cell line	IC_50_ (*μ*M)
C6 glioma cells	64.9 ± 1.3
MDA-MB-231 cells	53.3 ± 0.8
A549 cells	>100
