# Supplementary material for: Antiproliferative and Apoptosis-Inducing Activities of 4-Isopropyl-2,6-bis(1-phenylethyl)phenol Isolated from Butanol Fraction of Cordyceps bassiana
Source: Evid Based Complement Alternat Med. 2015 Apr 2;2015:739874. doi: 10.1155/2015/739874 (PMC4397031; doi:10.1155/2015/739874)

Supplementary Fig. 1

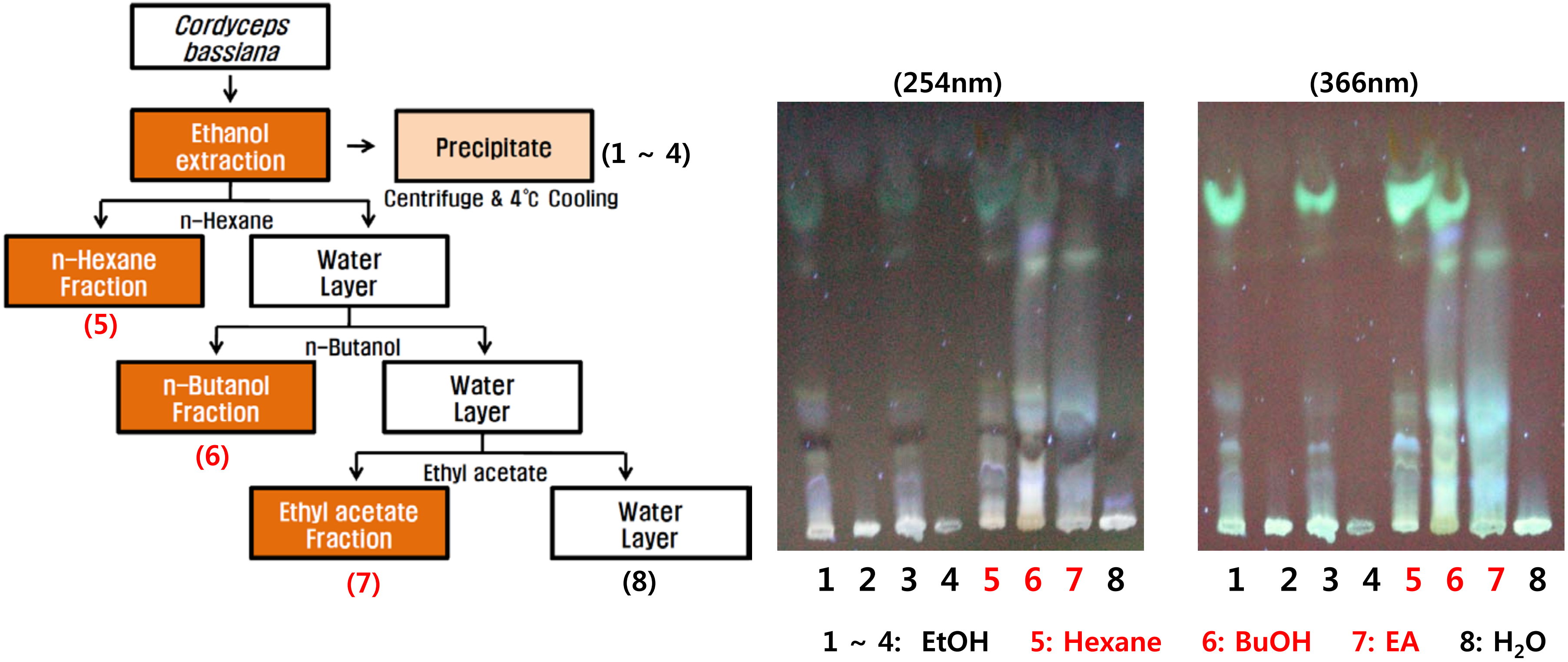

Supplementary Fig. 2

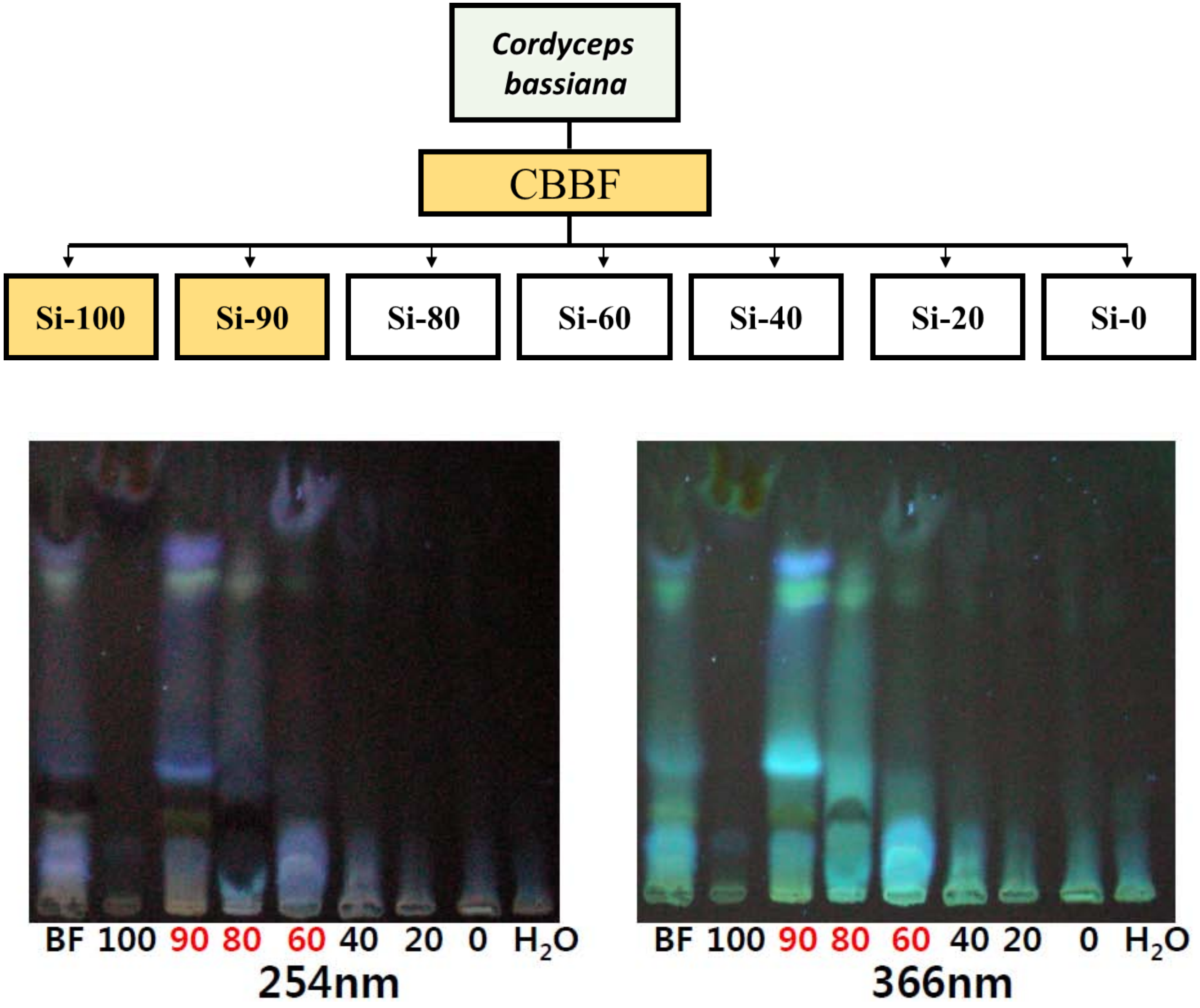

Supplementary Fig. 3

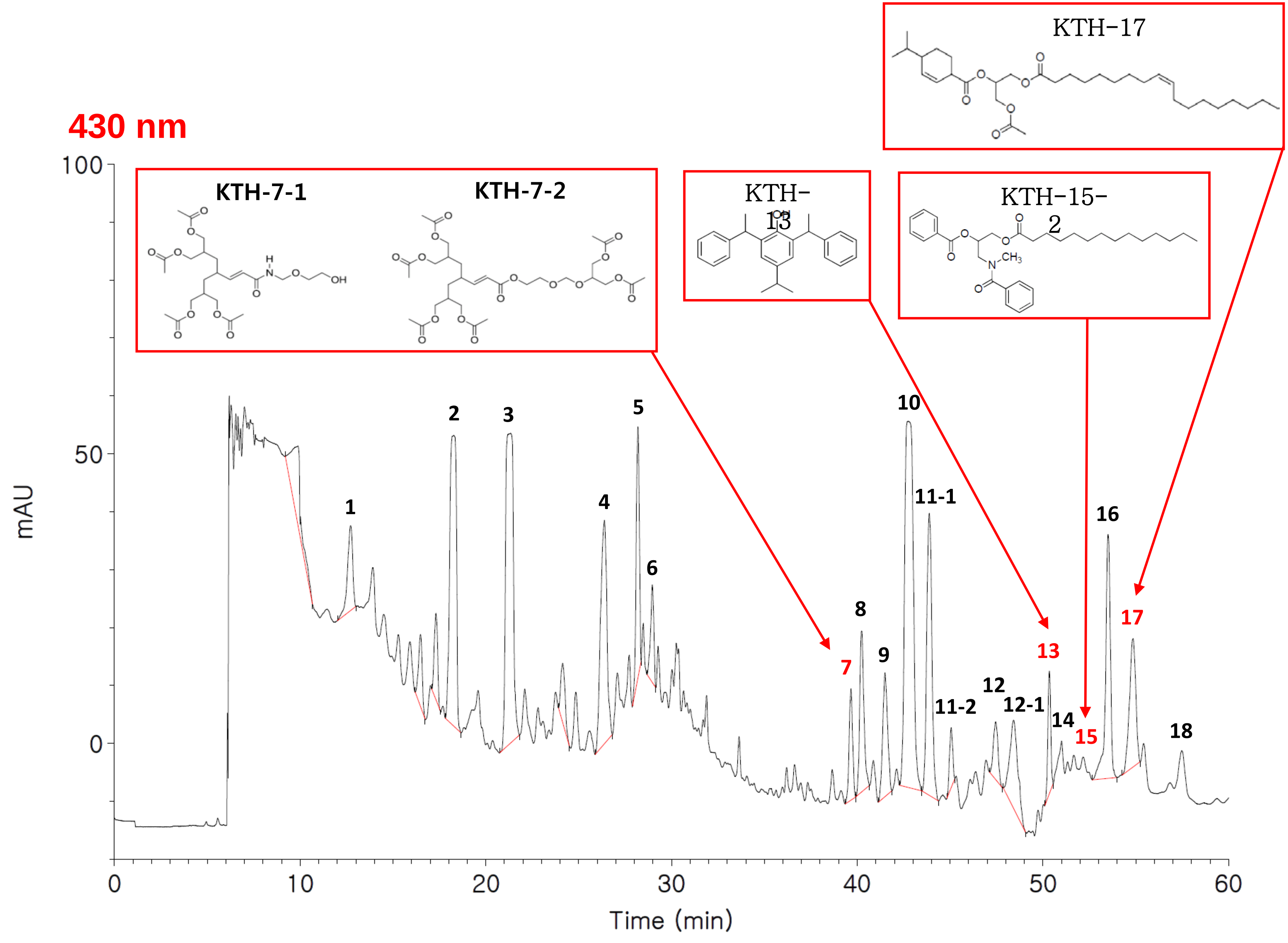

Supplementary Fig. 4

(A)

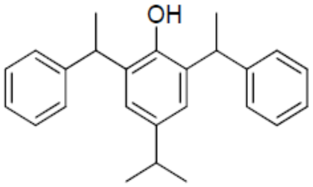

KTH-13 (M.W. = 344)

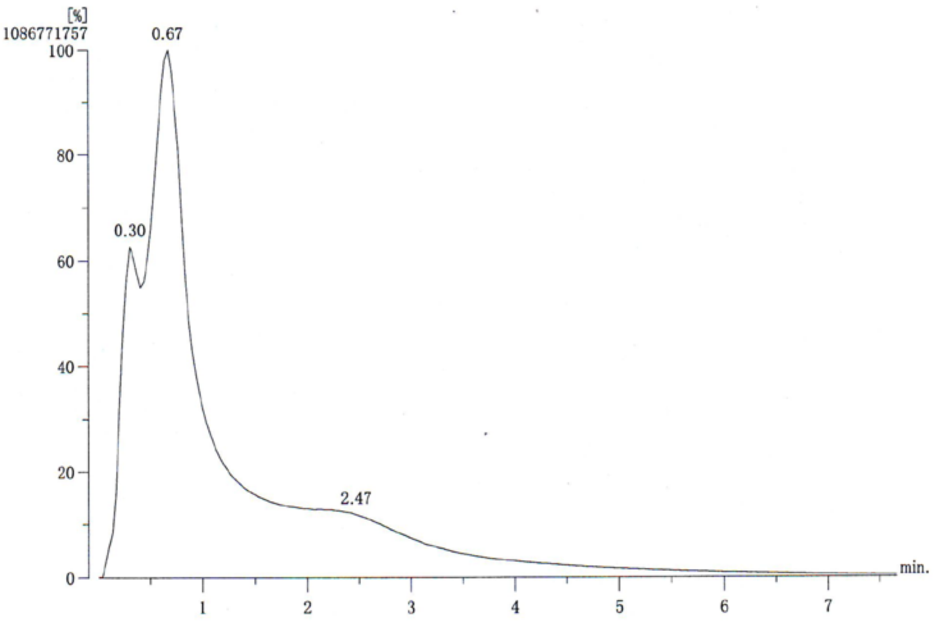

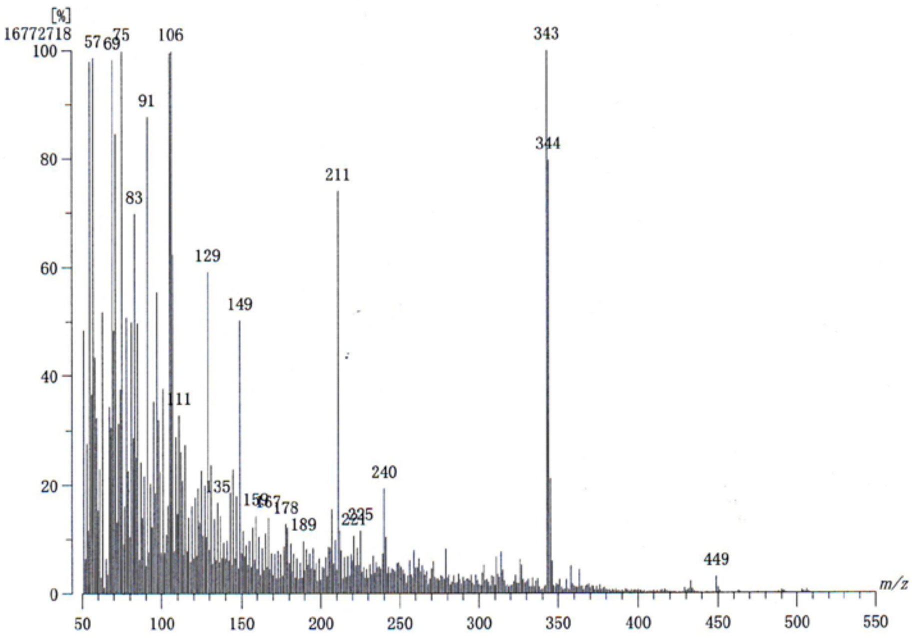

Supplementary Fig. 4

(B)

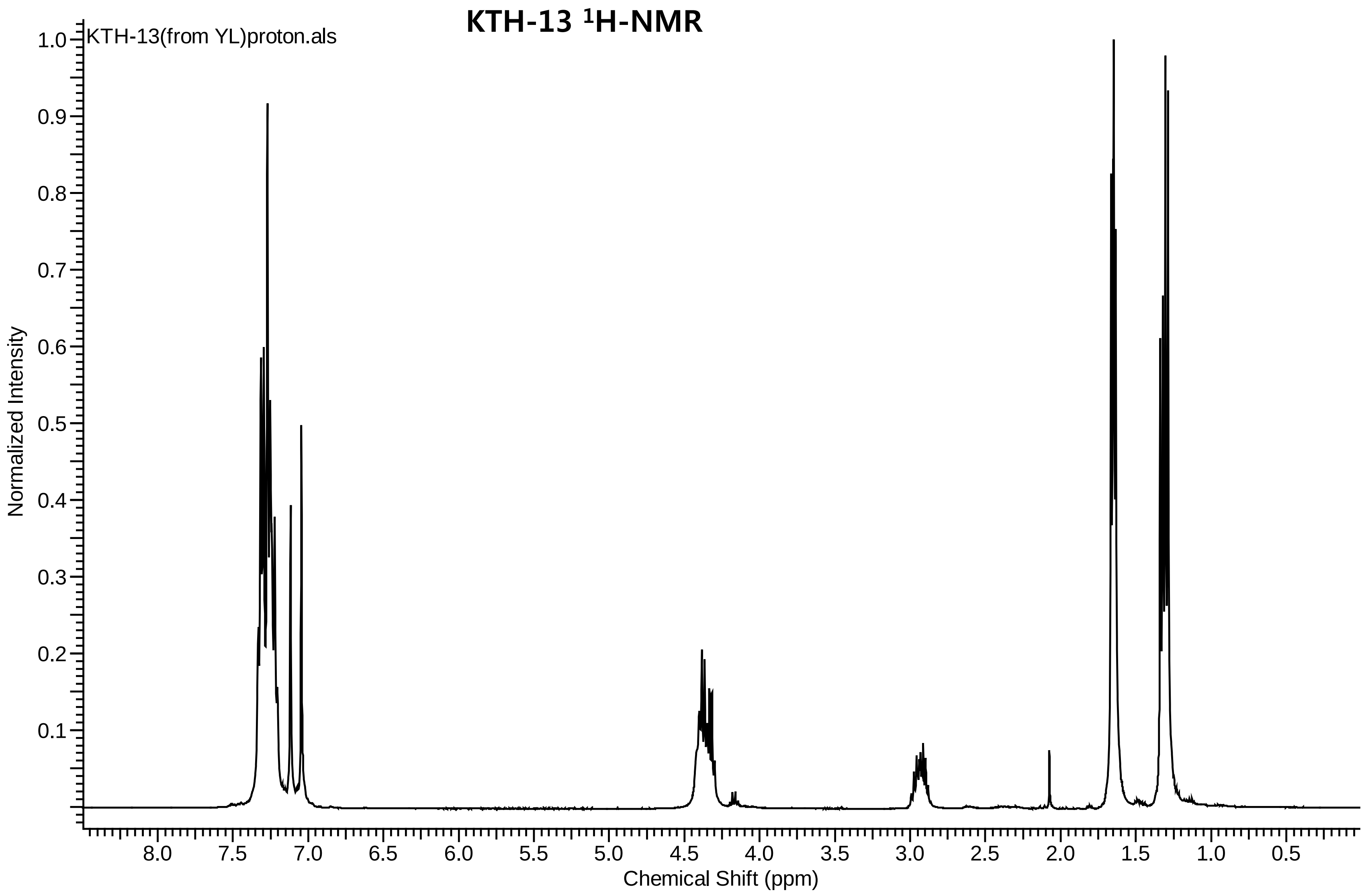

Supplementary Fig. 5

KTH-7-1
(M.W. = 502)

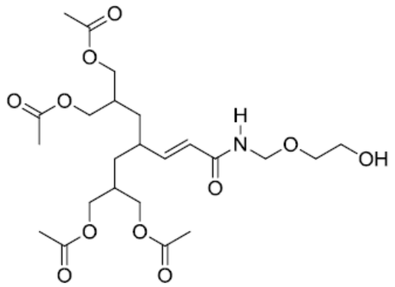

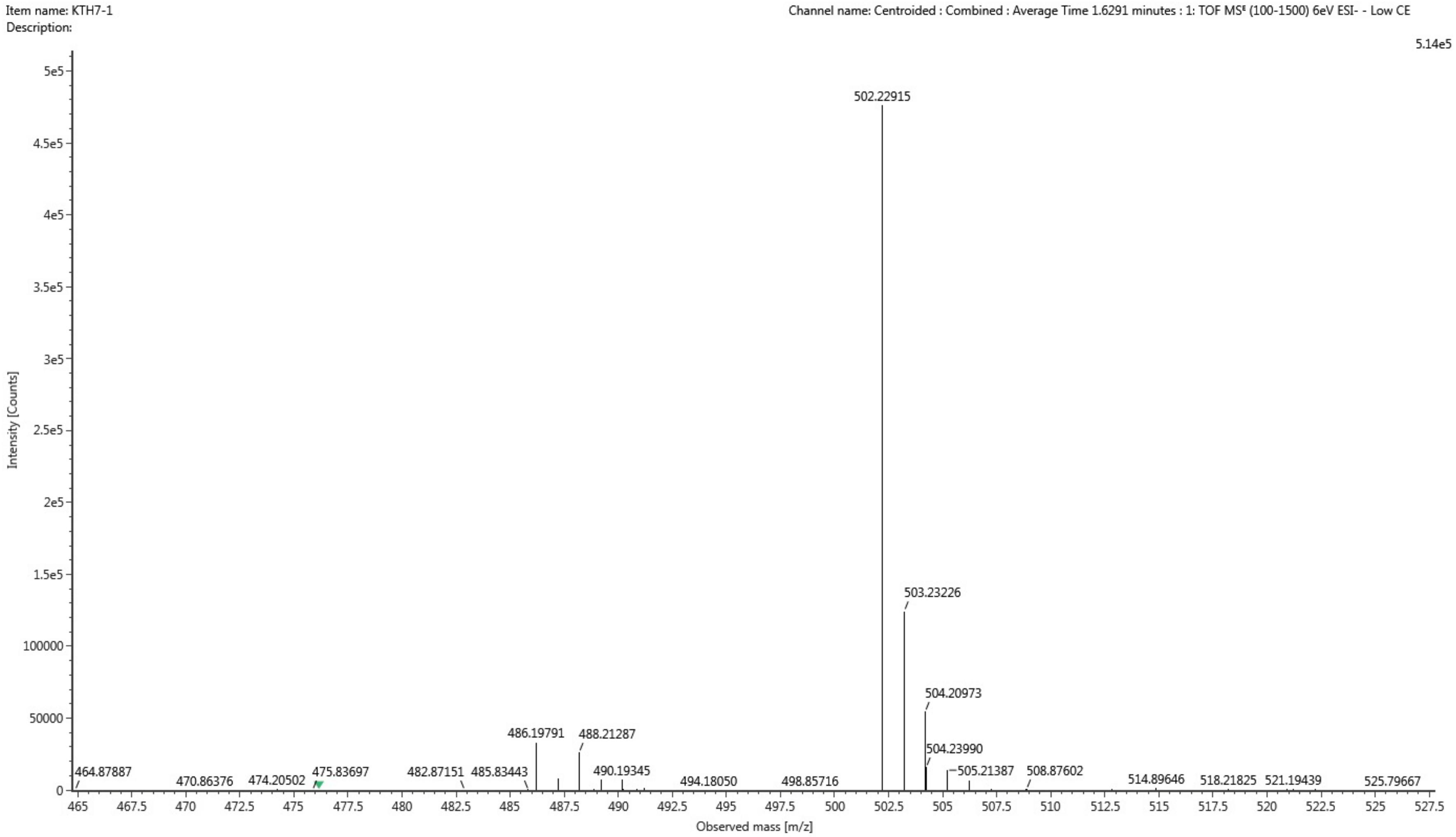

Supplementary Fig. 6

KTH-7-2
(M.W. = 663)

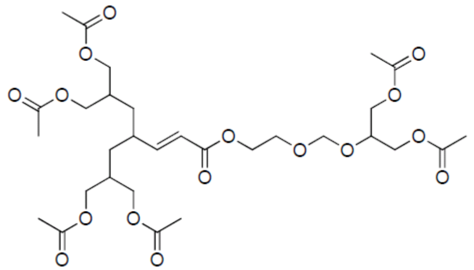

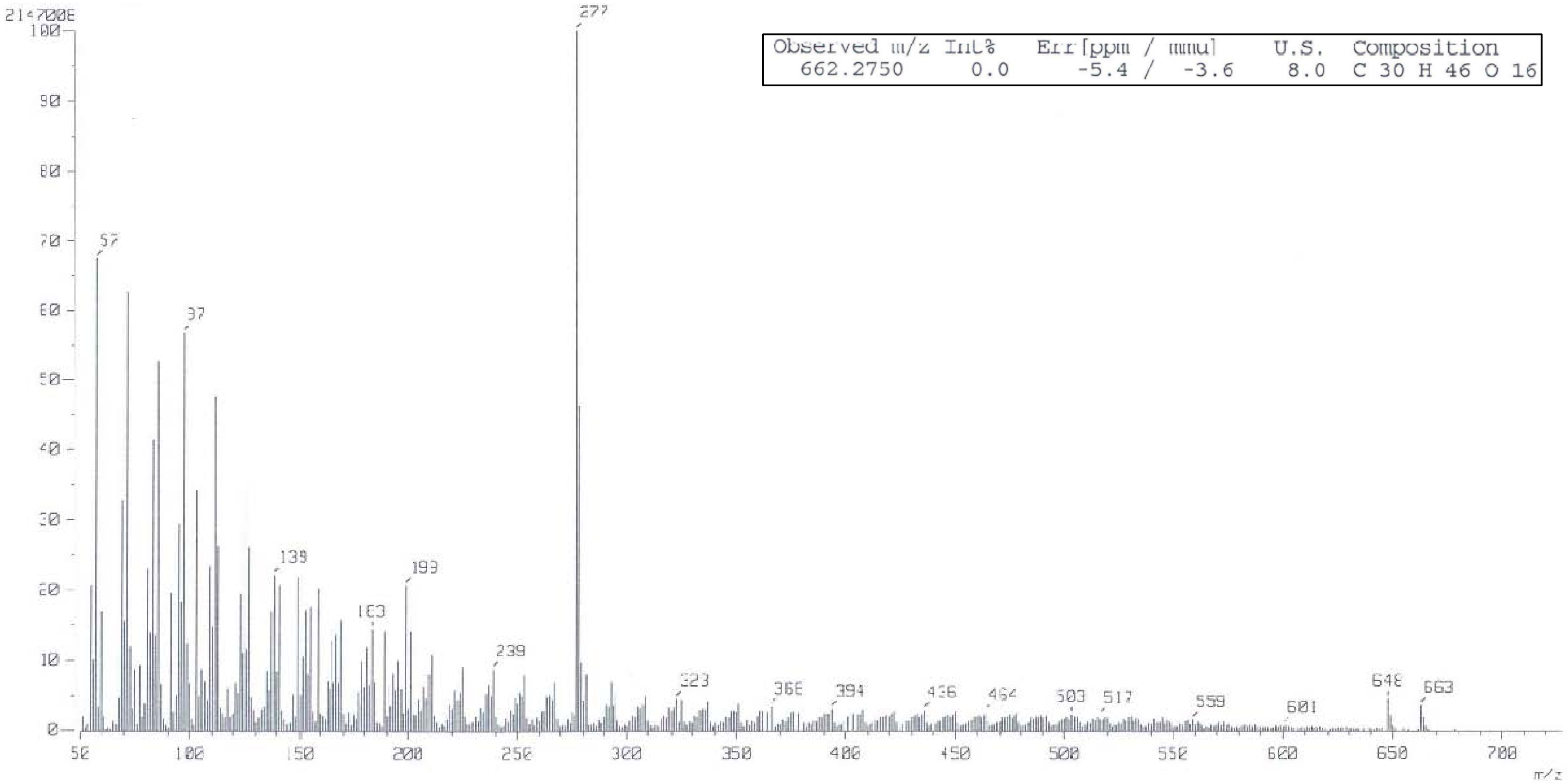

Supplementary Fig. 7

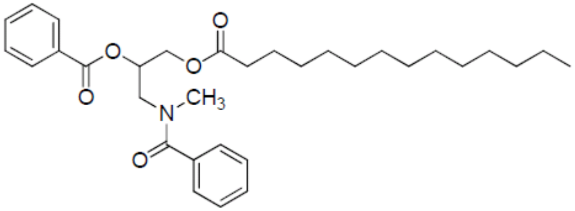

KTH-15-2 (M.W. = 523)

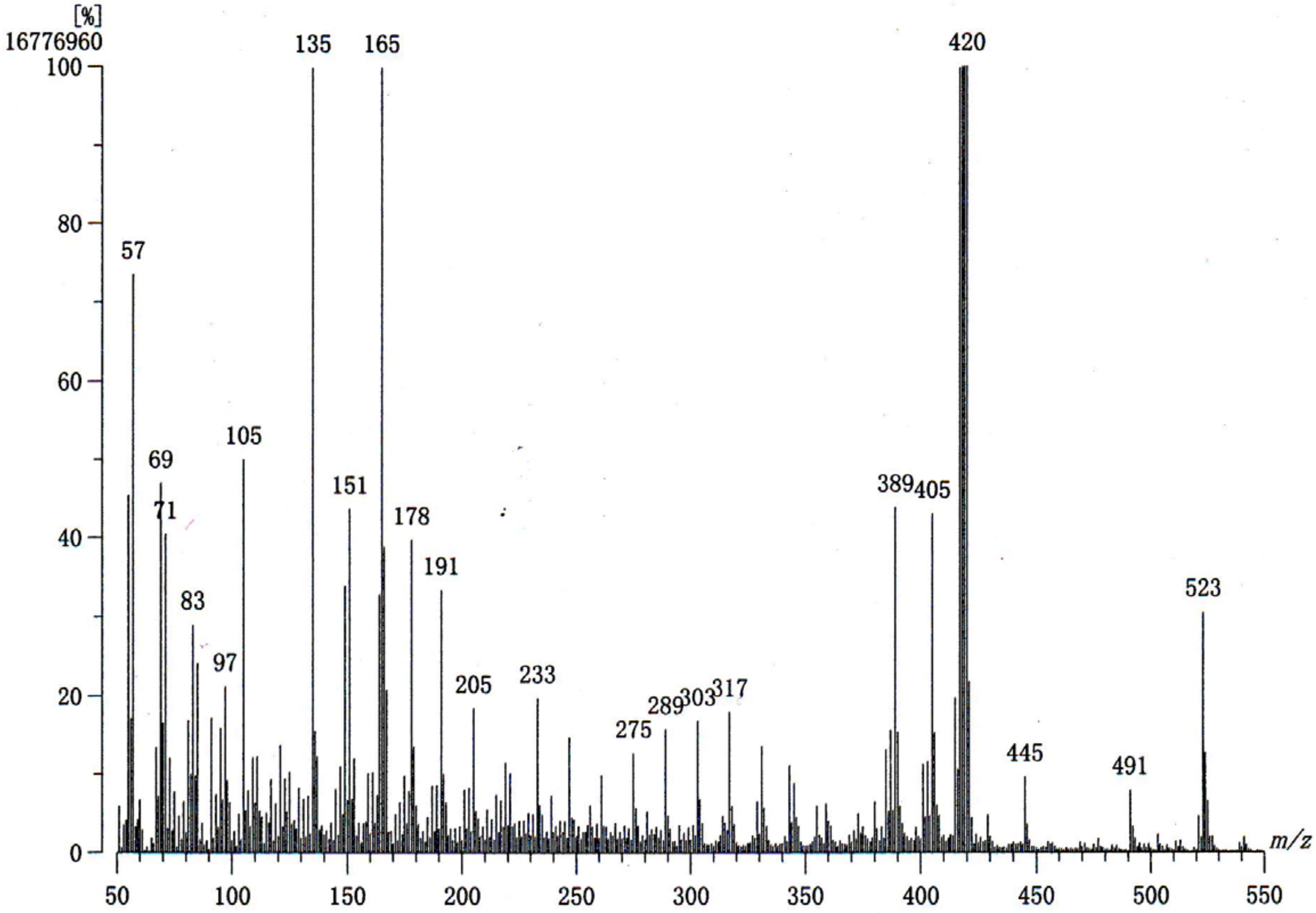

Supplementary Fig. 8

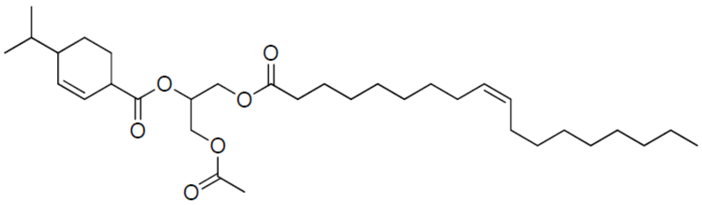

KTH-17 (M.W. = 506)

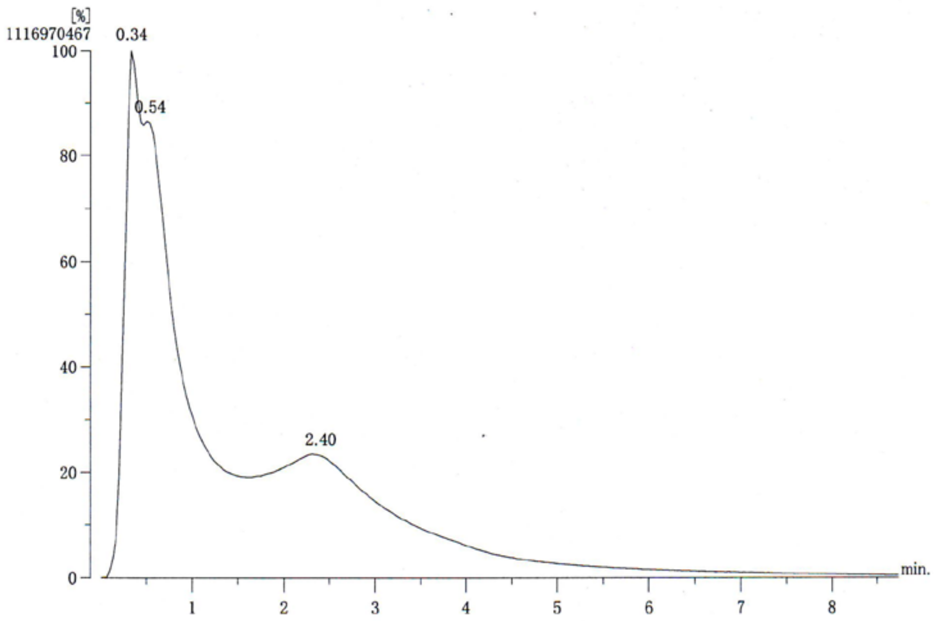

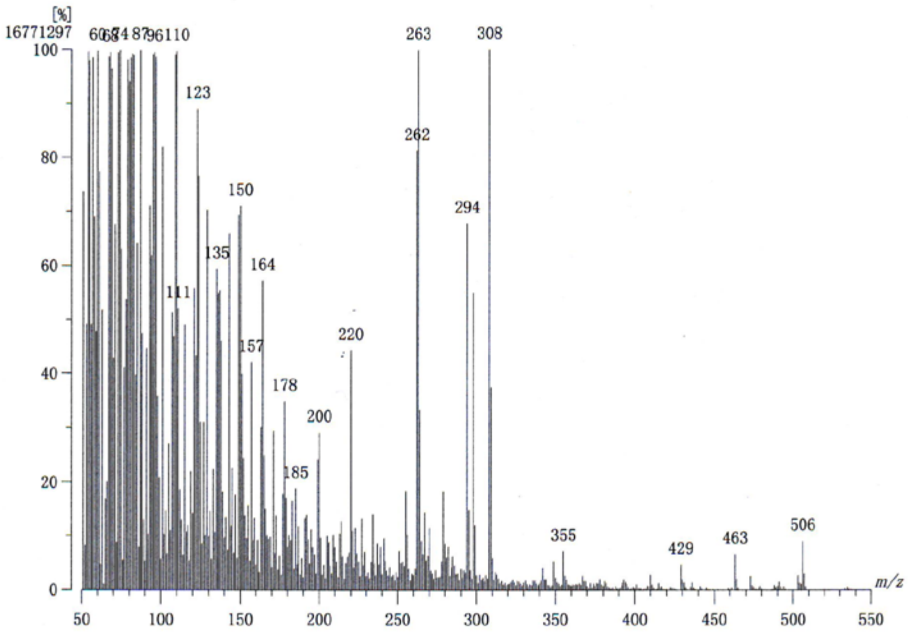

Supplement: Supplementary file 1 — Supplementary Fig. 1. Fractionation procedure to prepare solvent fractions by sequential solvent fractionation and thin layer chromatography profiles of C. bassiana ethanol extract (1) and its sub-fractions (5 to 8). The crude ethanol extract was subfractinated by sequential fractionation procedure with various solvents to afford eight sub-fractions. Ingredients in these fractions were also detected by thin layer chromatography. Supplementary Fig. 2. Fractionation procedure to prepare fractions by silica column chromatography and thin layer chromatography profiles of these fractions at 254 and 366 nm. The fractions were chromatographed over a silica gel column to afford seven sub-fractions. Ingredients in these fractions were detected by thin layer chromatography. Supplementary Fig. 3. Isolated compounds from peaks separated by prep-HPLC. Ingredient compounds displaying each peak in each fraction were purified by preparative HPLC. The structure of isolated compounds in each peak was identified by spectroscopic analysis. Supplementary Fig. 4. Spectroscopic profile of KTH-13. (A) Mass spectrum of KTH-13. Electrospray ionization tandem mass spectrometry (ESI MS) of KTH-13 was measured on an Agilent 1100 liquid chromatography/mass spectrometry (LC/MS) spectrometer with a Phenomenex Luna C18 analytical column. (B) 1H-NMR profile of KTH-13. 1H-NMR spectra of KTH-13 were recorded on a Bruker Avance 300 (300 MHz) and Bruker DPX 400 (400 MHz). Chemical shifts are reported in parts per million (ppm) downfield relative to tetramethylsilane as an internal standard. Supplementary Fig. 5. Mass spectrum of KTH-7-1. ESI MS of KTH-7-1 was measured on an Agilent 1100 LC/MS spectrometer with a Phenomenex Luna C18 analytical column. Supplementary Fig. 6. Mass spectrum of KTH-7-2. ESI MS of KTH-7-2 was measured on an Agilent 1100 LC/MS spectrometer with a Phenomenex Luna C18 analytical column. Supplementary Fig. 7. Mass spectrum of KTH-15-2. ESI MS of KTH-15-2 was measured on an Agilent [file 739874.f1.pdf]
